# Pediatric High Grade Glioma: a Review and Update on Tumor Clinical Characteristics and Biology

**DOI:** 10.3389/fonc.2012.00105

**Published:** 2012-08-24

**Authors:** Jason Fangusaro

**Affiliations:** Pediatric Neuro-Oncology, The Ann & Robert H. Lurie Children’s Hospital of Chicago, Feinberg School of Medicine, Northwestern UniversityChicago, IL, USA

**Keywords:** pediatric, high grade glioma, review

## Abstract

High grade gliomas (HGG) are one of the most common central nervous system (CNS) tumors encountered in adults, but they only represent approximately 8–12% of all pediatric CNS tumors. Historically, pediatric HGG were thought to be similar to adult HGG since they appear histologically identical; however, molecular, genetic, and biologic data reveal that they are distinct. Similar to adults, pediatric HGG are very aggressive and malignant lesions with few patients achieving long-term survival despite a variety of therapies. Initial treatment strategies typically consist of a gross total resection (GTR) when feasible followed by focal radiotherapy combined with chemotherapy. Over the last few decades, a wealth of data has emerged from basic science and pre-clinical animal models helping to better define the common biologic, genetic, and molecular make-up of these tumors. These data have not only provided a better understanding of tumor biology, but they have also provided new areas of research targeting molecular and genetic pathways with the potential for novel treatment strategies and improved patient outcomes. Here we provide a review of pediatric non-brainstem HGG, including epidemiology, presentation, histology, imaging characteristics, treatments, survival outcomes, and an overview of both basic and translational research. An understanding of all relevant pre-clinical tumor models, including their strengths and pitfalls is essential in realizing improved patient outcomes in this population.

## INTRODUCTION

High grade gliomas (HGG) represent one of the most common central nervous system (CNS) tumors among adults. This contrasts significantly to the pediatric population where HGG only comprise approximately 8–12% of all primary CNS tumors (Bondy et al., 2008). In adults, HGG often arise from a low grade glioma (LGG) that has undergone malignant transformation, but this phenomenon is exceedingly rare in pediatric patients (Broniscer et al., 2007). Similar to the adult experience, however, pediatric HGG are characterized by their aggressive clinical behavior and account for a significant amount of morbidity and mortality among children with brain tumors. HGG typically arise from astrocytic origins, including glial, oligodendrocytes, and ependymal cells (Louis et al., 2007). These tumors are classified by the World Health Organization (WHO) as either grade III or IV meaning that they are highly malignant tumors with characteristic findings such as hypercellularity, nuclear atypia, and high mitotic activity with or without microvascular proliferation and pseudopalisading necrosis (Kleihues et al., 2007; Louis et al., 2007). HGG include a variety of heterogeneous lesions with differing histologies, but the most common histologies are anaplastic astrocytoma (WHO Grade III) and glioblastoma multiforme (GBM; WHO grade IV). Despite numerous treatment approaches, outcomes have remained dismal with most series showing 5-year survival outcomes ranging from 15 to 35% and the far majority of children succumbing to their disease (Broniscer and Gajjar, 2004; Finlay and Zacharoulis, 2005; Broniscer, 2006; Cohen et al., 2011). Although there truly is no one accepted standard of care and treatment algorithms can vary, most experts agree that a gross total resection (GTR) followed by focal irradiation to the tumor bed plus additional chemotherapy is an appropriate treatment approach (Fangusaro, 2009; Jones et al., 2012).

As with most tumor types, one proposed key to improving survival outcomes is a better understanding of tumor biology. Since pediatric HGG histologically resemble adult HGG, historically, it was believed that these were similar tumors. New biologic, molecular, and genetic data suggest that pediatric HGG are distinct from adult HGG (Jones et al., 2012). In fact, over the last few decades, significant data has emerged greatly enhancing our understanding of pediatric HGG biology. These data have given rise to unique research opportunities that can exploit our biologic knowledge of these tumors in an effort to utilize novel biologically targeted agents with the potential to improve patient survival. Here we present an overview of non-brainstem pediatric HGG and review the basic epidemiology, presentation, histology, imaging characteristics, treatment strategies, and survival outcomes. We also review the current understanding of pediatric HGG biology and current models utilized to assess these tumors.

## EPIDEMIOLOGY AND ETIOLOGY

According to the Central Brain Tumor Registry of the United States (CBTRUS) for the years 2004–2008, approximately 7% of all reported brain tumors occurred in children 0–19 years old (y/o). Among children 0–19 y/o the overall total incidence of HGG (including anaplastic astrocytoma, anaplastic oligodendroglioma, glioblastoma, mixed glioma, and malignant glioma) is approximately 0.85 per 100,000 (CBTRUS, 2012). Most series estimate that HGG represent approximately 8–12% of all childhood CNS tumors and the distribution between males and females is relatively equal (Finlay and Zacharoulis, 2005; Broniscer, 2006). Although they can occur anywhere within the CNS, the most common location is within the supratentorial compartment when brainstem lesions are excluded (Broniscer and Gajjar, 2004). It is estimated that 35–50% are located within the cerebral hemispheres with a smaller percentage emanating from the thalamus, hypothalamus, third ventricle, and basal ganglia. Primary spinal cord lesions are much less common with an incidence of approximately 3% in children (Wolff et al., 2012). Non-brainstem infratentorial lesions are most commonly seen in younger children as compared to adolescents and young adults (Fangusaro, 2009). The highest incidence of supratentorial HGG among children is in patients 15–19 y/o with a median age of approximately 9 y/o; however, HGG can be seen in any age group starting from *in utero* and early infancy through young adulthood suggesting multiple contributing factors to their etiology (Seker and Ozek, 2006; Hou et al., 2008; Milano et al., 2009).

A variety of hypotheses have been postulated in an effort to more fully understand the etiology of pediatric CNS tumors, including HGG, but the cause of the majority is greatly unknown. There are a few established risk factors that predispose children to the development of HGG. One well understood risk factor is exposure to ionizing radiation, typically for the treatment of a previous oncologic condition, such as acute leukemia. A study performed at St. Jude Research Children’s Hospital identified a dose-dependent effect on tumor development from previous radiation exposure (Walter et al., 1998). The study also concluded that children who received radiation before the age of 6 y/o had the highest risk of developing a secondary malignancy (Walter et al., 1998). These findings were further supported in a larger subsequent cohort from the Childhood Cancer Survivor Study (Neglia et al., 2006). Most other exposures thought possibly related to brain tumor development (cell phone use, infections, trauma, and toxins) have not consistently been shown statistically related to brain tumor development suggesting that the true etiology is most likely multifactorial (Baldwin and Preston-Martin, 2004).

There are also rare genetic diseases that predispose a child to the development of a HGG. Most of these are inherited defects in the regulation of cell proliferation and apoptosis typically caused by germline mutations (Melean et al., 2004). Neurofibromatosis type I is an autosomal recessive disorder and the most common inherited genetic disorder predisposing children and adults to CNS tumor development. A mutation in the *NF-1* gene results in an absence of a protein called neurofibromin. Normally, this protein regulates growth and *Ras*, a proto-oncogene, so in its absence, unregulated cell growth and oncogenesis can ensue (Ward and Gutmann, 2005). Far and away, these patients have an increased risk of developing LGG within the CNS, typically the optic pathway, but there are data showing that these patients are also at an increased risk for developing HGG (Rosenfeld et al., 2010). Another disorder associated with HGG development is Li-Fraumeni syndrome. In Li Fraumeni, patients exhibit a defect in *TP53* gene which encodes for the checkpoint protein, p53. *TP53* normally acts as a tumor suppressor gene by inducing pathways that cause cell cycle arrest, apoptosis, and inhibit angiogenesis (Melean et al., 2004). A mutation in this system leads to unregulated cell proliferation and an increased risk of malignant transformation. These patients can develop a variety of malignancies, typically at a younger age, including HGG (Varley, 2003). Other rare genetic disorders that increase the risk of CNS tumor development include Turcot’s syndrome, Tuberous sclerosis, and von Hippel–Lindau disease (Hamada et al., 1998; Varley, 2003; Melean et al., 2004). Patients with Turcot’s syndrome typically have a defect in the adenomatous polyposis coli (APC) gene and/or a mutation in DNA mismatch repair (MMR) genes predisposing them to the development of multiple colorectal adenomas, colorectal adenocarcinoma, and primary brain tumors (Itoh et al., 1993; Melean et al., 2004). The MMR mutations are thought to be associated with the development of HGG in these patients whereas the APC defects are more closely associated with medulloblastoma development (Melean et al., 2004). Although tuberous sclerosis and von Hippel–Lindau disease both predispose patients to CNS tumor development, these patients typically do not develop HGGs. These genetic disorders have contributed greatly to our understanding of tumor biology and development; however, they can be linked to only a fraction of HGG cases in children with the remainder (and majority) of cases having no known identifiable cause.

## CLINICAL PRESENTATION, DIAGNOSIS, AND PROGNOSTIC FACTORS

Children presenting with a new diagnosis of a HGG often develop the same symptoms common to many newly diagnosed CNS tumors. These presenting signs are often due to increased intracranial pressure including persistent headaches, behavior changes, early morning nausea/emesis, diplopia, and papilledema. Patients may also present with more specific localizing symptoms such as focal motor deficits, hemiplegia, pyramidal tract findings, dysmetria, and chorea depending upon the tumor’s location (Fangusaro, 2009). As compared to LGG, the typical duration of symptoms prior to presentation is often much shorter in children with HGG. This is hypothesized to be due to the increased mitotic activity and faster growth rate of these tumors leading to more rapid invasion of the adjacent normal brain tissue (Reddy and Wellons, 2003; Reulecke et al., 2008). Although patients with HGG can develop seizures, this is not a common presentation at diagnosis. Seizures in the setting of a HGG often occur when the tumor invades the temporal lobe, a common seizure focus. Seizures are a much more common presentation in specific low grade CNS tumors such as ganglioglioma and dysembryoplastic neuroepithelial tumors (DNET; Khajavi et al., 1995; Weissman et al., 1996). As compared to older children, infants and young children often present with non-specific findings such as failure to thrive, lethargy, nausea/emesis, and macrocephaly often making the diagnosis difficult as many of these symptoms may go unnoticed or are attributed to other common childhood illnesses, such as a viral infection (Reddy and Wellons, 2003).

The first diagnostic tool of choice in most children suspected of having an intracranial process is a non-contrast computerized tomography (CT) scan. This imaging technique is quick, often eliminating the need for sedation in young children, and it is a good screening tool to evaluate for hydrocephalus and acute CNS hemorrhage. CT may also help identify a space-occupying lesion/mass. As with most pediatric CNS tumors, however, magnetic resonance imaging (MRI) is the imaging modality of choice. MRI not only provides a clearer picture regarding tumor location and invasion into surrounding brain, but it is an essential tool necessary for neurosurgical and radiation planning. Although there is no one specific MRI finding that can distinguish a HGG from other pediatric CNS tumors, there are some characteristics that are common to HGG that may help guide the differential diagnosis. Typically, HGG have less distinct and irregular borders as compared to other CNS tumors. They may have mass effect on surrounding brain structures and nodular enhancement. Classically, these lesions are hypo-intense on T1- and hyper-intense on T2-weighted MRI sequences and induce significant edema as noted on fluid attenuation inversion recovery (FLAIR) images (Panigrahy and Bluml, 2009). Although contrast-enhancement is commonly seen in supratentorial HGG, the amount and degree of enhancement does not always correlate to tumor grade (Warren, 2008). Most HGGs are locally invasive and rarely present with distant metastases or leptomeningeal spread (Broniscer and Gajjar, 2004; Fangusaro, 2009). Therefore, imaging of the spinal axis in patients with intracranial lesions is physician dependent, but many clinicians will obtain a complete spine MRI at diagnosis as a baseline or if specific symptoms warrant assessment.

Advanced imaging techniques are currently being researched in an effort to help guide diagnosis and treatment strategies. For example, perfusion-weighted MRI is a non-invasive technique that helps determine tumor angiogenesis and capillary permeability. HGG commonly demonstrate increased blood flow to a tumor lesion demonstrated on this modality (Panigrahy and Bluml, 2009). Magnetic resonance spectroscopy (MRS) is another modality that is being increasingly evaluated. MRS utilizes imaging to understand the metabolic profile of a specific area of interest within the brain. In gliomas, as the tumor grade increases to a more malignant variant, there is an increase in the choline to *N*-acetyl aspartate (NAA) ratio reflecting the increased metabolic activity present in higher grade lesions (Lemort et al., 2007; Panigrahy and Bluml, 2009). These are just a couple of the many ongoing advanced imaging techniques being studied. None of the advanced imaging techniques evaluated thus far can conclusively diagnose a specific histology, and therefore, a biopsy or surgical resection is recommended whenever possible to establish a diagnosis.

Surgical resection is important for many reasons, including establishing a diagnosis of HGG, relieving intracranial pressure, and contributing to prognosis. Independent from tumor location and specific histology, the amount of surgical resection is one of the most important clinical prognostic factors identified to date in children with supratentorial HGG (Finlay et al., 1995; Broniscer and Gajjar, 2004). The Children’s Cancer Group (CCG) study-945 showed that those children with HGG who underwent a surgical resection of 90% or greater had a progression-free survival (PFS) of 35 ± 7% as compared to a 5-year PFS of 17 ± 4% in patients who did not (Finlay et al., 1995). Therefore, every attempt should be made at a complete surgical resection when safe and feasible in an effort to maximize patient survival. Unfortunately, complete resection may not be possible in many cases, especially in patients harboring tumors invading critical structures, those with midline tumors and infratentorial tumors involving the cerebellum and brainstem. Histologic grade has proven prognostic in some series whereas those patients with a WHO grade III tumor have improved survivals as compared to those with WHO grade IV tumors. In the aforementioned CCG-945 trial, patients with an anaplastic astrocytoma (WHO grade III) had statistically improved survival with estimated 5-year PFS and overall survival (OS) of 28 and 29%, respectively, as compared to patients with GBM (WHO grade IV) who had a 5-year PFS and OS of 16 and 18%, respectively (Finlay et al., 1995).

The CCG-945 trial also looked at a variety of molecular and cytogenetic markers in an effort to better define prognostic variables in pediatric HGG. An analysis of p53 revealed that those patients with overexpression of p53 and/or a mutation in the *TP53* gene had significantly lower PFS as compared to children who had neither of these findings. Abnormalities of p53 were most commonly seen in WHO grade IV tumors; however, p53 was shown to be an independent prognostic factor regardless of histologic grade (Pollack et al., 2002). A follow-up analysis of O6-methylguanine-DNA methyltransferase (MGMT) status in this same group of patients revealed a statistically worse outcome in children with overexpression of MGMT (Pollack et al., 2006).

Interestingly, there appears to be a subset of younger children who harbor histologically proven HGG that have a more indolent course as compared to older children. It is believed that the biology of these tumors is distinct despite the histologic similarities. There is data in a small number of cases showing that infant HGG appear to lack HOXA9/HOXA10 which are thought important for self-renewal (Jones et al., 2012). HOXA9/HOXA10 have been associated with more malignant variants with poor prognosis in some adults and older children with HGG (Jones et al., 2012). This may help explain why these young children with HGG have seemingly less aggressive disease. In a prospective French study evaluating the use of chemotherapy in young children less than 5 y/o with newly diagnosed HGG, 5-year PFS was 35% and 5-year OS was 59%, with a median follow-up of 5.2 years (Dufour et al., 2006). In a separate retrospective study performed at St. Jude Research Hospital reviewing the clinical characteristics and survival in children under 3 y/o with HGG, 5-year event-free survival (EFS) and OS were 28.6 and 66.3%, respectively (Sanders et al., 2007). These outcomes are far superior to those published in older children with HGG again suggesting that these tumors may be distinct (Finlay et al., 1995; Broniscer and Gajjar, 2004; Cohen et al., 2011). Other groups have shown this same phenomenon whereby younger children have an improved survival outcome even when utilizing radiation-sparing treatment strategies (Geyer et al., 1995; Duffner et al., 1996). It is unclear, however, if it truly is a specific age that is prognostic or if younger children simply develop HGG tumors that have unique biologic and molecular characteristics that confer a better prognosis.

## TREATMENT STRATEGIES AND OUTCOMES

The initial treatment strategy for a child with a newly diagnosed HGG is to attempt a maximal safe surgical resection. This recommendation is based upon the data from the previously mentioned CCG-945 showing that amount of surgical resection is a prognostic variable (Finlay et al., 1995). Even when a complete radiographic GTR is achieved, it is understood that microscopic tumor cells are still present. This is due to the infiltrative nature of these lesions making it virtually impossible to achieve a GTR with clear surgical margins without risking significant morbidity (Broniscer and Gajjar, 2004; Fangusaro, 2009). Additional therapy is necessary in an attempt to prevent the high likelihood of local recurrence. Radiation therapy has become the mainstay of therapy, particularly for those children older than 3 y/o with newly diagnosed HGG. Since younger children are more susceptible to the negative deleterious effects associated with radiation therapy and they seem to harbor more indolent tumors, they are often treated with chemotherapy alone and radiation-sparing approaches (Geyer et al., 1995; Duffner et al., 1996; Dufour et al., 2006; Sanders et al., 2007). For older children, focal radiotherapy with a margin around the tumor bed has become the standard. Previous studies have shown that there is no role for whole brain radiotherapy in patients with localized HGG (Buckner et al., 2007). Typically, the conventional dosing for a child with a newly diagnosed HGG is 50–60 Gy delivered in daily fractions of approximately 180–200 cGy over a 6 week period. Alternative radiotherapy techniques such as hyper- and hypo-fractionation have not consistently proven to be statistically beneficial in children with HGG and are typically not utilized outside of a clinical trial setting (Fallai and Olmi, 1997).

Chemotherapy was first introduced into the treatment schema for children with newly diagnosed HGG in the 1970s. Despite a few publications reporting the additional benefit to survival as compared to radiation therapy alone, its exact role and true survival benefit remain disputed. In the CCG-943 trial, children with newly diagnosed HGG were randomized to receive either focal radiation therapy alone to a dose of 54 Gy or the same radiotherapy with a combination of concomitant and maintenance chemotherapy. Patients randomized to receive chemotherapy received weekly vincristine during radiation followed by eight maintenance chemotherapy cycles consisting of prednisone, lomustine, and vincristine (pCV) each given approximately 6 weeks apart (Sposto et al., 1989). Five-year EFS was 46% in the chemotherapy treated group versus 18% in the radiation alone group which was a statistically significant difference; however, a central pathology review performed many years later revealed that many of the patients included in this study harbored LGGs (Sposto et al., 1989; Finlay and Zacharoulis, 2005). Despite this, there still appeared to be a statistical benefit with the addition of chemotherapy to radiotherapy in patients with GBM (WHO grade IV). Since this trial, the addition of chemotherapy to radiotherapy has been adopted by many as the accepted “standard of care” for children with newly diagnosed HGG. Unfortunately, numerous subsequent combination studies completed over the last 40 years have never reached the outcomes achieved by the CCG-943 trial, suggesting that the addition of LGGs in this cohort skewed the survivals reported.

The immediate successor to CCG-943 was the CCG-945 trial. In CCG-945, children with HGG were randomized to one of two chemotherapy regimens in addition to focal radiotherapy. The conventional arm was the same chemotherapy given in the CCG-943 trial (pCV) and the experimental arm was the so called “8 in 1” regimen, a combination of eight agents all given within a short time period (prednisone, lomustine, vincristine, hydroxyurea, cisplatin, cytarabine, dacarbazine, and procarbazine). Patients assigned to the “8 in 1” arm received two cycles of pre-radiation chemotherapy, and those patients less than 2 y/o were non-randomly assigned to the “8 in 1” regimen. There was no statistical difference between the two arms and the outcomes were worse as compared to the previous CCG-943. Five-year PFS was 26 ± 8% in the conventional arm versus 33 ± 8% in the experimental arm (Finlay et al., 1995).

There have been a variety of trials conducted in pediatric patients utilizing a diverse group of biologic and chemotherapeutic agents in combination with focal radiotherapy. All have had similar survival results, again never achieving the outcomes reported in the original CCG-943 trial. In 2005, Stupp and colleagues published data showing that the addition of temozolomide to radiotherapy for newly diagnosed GBM in adult patients resulted in a clinically meaningful and statistically significant survival benefit with minimal additional toxicity as compared to radiation alone (Stupp et al., 2005). This agent was an oral alkylating agent and overall well tolerated, so many experts had high hopes for its application in the pediatric HGG population. The adult trial prompted the development of a pediatric trial, the Children’s Oncology Group (COG) ACNS-0126 study. ACNS-0126 was a phase II trial whereby children with newly diagnosed HGG received daily temozolomide during radiotherapy followed by maintenance temozolomide. There was not a randomization to radiation alone, since the previous CCG-943 trial had already established a benefit utilizing chemotherapy. Temozolomide showed no survival benefit as compared to historic controls (Cohen et al., 2011). In truth, the pediatric trial did not ask the same question as the successful adult trial comparing to radiation alone, but the results were still no better than the numerous preceding pediatric HGG trials. Despite this, the improved tolerability and ease of administration have lead many clinicians to continue to utilize this strategy when treating newly diagnosed patients who are not enrolled on a clinical trial (Fangusaro and Warren, 2012).

There has also been an attempt to overcome the resistance to alkylators apparent in some patients with HGG. For example, the disappointing responses to temozolomide observed in children with HGG are in part thought attributable to overexpression of DNA repair proteins, particularly MGMT (Donson et al., 2007). This has led to clinical trials attempting to overcome this resistance in an effort to achieve a therapeutic response. In a study performed by the Pediatric Brain Tumor Consortium (PBTC), pediatric patients with recurrent or progressive HGG were treated with the combination of O6-benzylguanine (O6BG) and temozolomide. Forty-one patients were evaluable for response, including 25 patients with HGG. Although the combination was tolerable, it did not achieve the target response rate for activity (Warren et al., 2012). This population of patients with recurrent HGG has proven exceedingly difficult to treat with very few treatment options providing clinically meaningful responses.

There are fewer options and an even worse prognosis for children with recurrent HGG, with almost all children succumbing to their disease. One approach to treating children with recurrent HGG has been the use of high dose chemotherapy followed by autologous hematopoietic cell rescue. Although this is still considered controversial and is not universally accepted, the literature does suggest there may be a role for this strategy in a specific group of children with recurrent disease (Guruangan et al., 1998; Finlay et al., 2008). In a study by Finlay et al. (2008), 27 children with recurrent malignant astrocytomas received myeloablative chemotherapy followed by autologous marrow rescue with thiotepa and etoposide-based chemotherapy regimens. Five of 27 children survived event-free from 8.3 to 13.3 years at the time of publication. Another study by Guruangan et al. (1998) evaluated the outcome of myeloablative chemotherapy and autologous bone marrow rescue with or without radiotherapy in children younger than 6 years of age with a variety of recurrent malignant brain tumors who had not previously received irradiation. Twenty patients with recurrent brain tumors were enrolled. Ten of 20 (50%) patients, including three patients with HGG were alive and disease free at a median of 37.9 months at the time of publication. They concluded that myeloablative chemotherapy with autologous hematopoietic cell rescue followed by additional external-beam irradiation appeared to be an effective retrieval therapy for some young children with recurrent brain tumors (Guruangan et al., 1998). These data suggest again that there may be a subgroup of children with recurrent HGG for which this strategy is appropriate.

In adult HGG, the use of bevacizumab (BVZ), an anti-angiogenic agent that blocks vascular endothelial growth factor (VEGF), has shown promising results and a survival benefit in patients with recurrent HGG (Narayana et al., 2009; Huylebrouck et al., 2012; Morris, 2012). These findings led to the development of a pediatric trial within the PBTC-022 utilizing BVZ and CPT-11 in children with recurrent CNS tumors, including a HGG and diffuse intrinsic pontine glioma (DIPG) strata (Gururangan et al., 2010). Thirty-one evaluable patients received a median of two courses of BVZ plus CPT-11. There were no sustained responses in either HGG or DIPG. Median time to progression was 127 days for HGG patients and 6-month PFS was 41.8%. Although the regimen was well-tolerated, it showed minimal efficacy in children with recurrent HGG (Gururangan et al., 2010).

Bevacizumab has also recently been evaluated in up-front studies for adults with newly diagnosed HGG. An adult feasibility study evaluating the use of BVZ given concurrently with radiation therapy and daily temozolomide revealed that the combination was feasible. Radiographic responses were noted in 13 of 14 assessable patients (Narayana et al., 2008). In another pilot Phase II study of BVZ in combination with temozolomide and regional radiation therapy for up-front treatment of adult patients with newly diagnosed GBM, the interim analysis of 10 patients reported that the observed toxicities were acceptable to continue enrollment toward the overall target group of 70 patients. Also, the preliminary efficacy analysis showed encouraging PFS (Lai et al., 2008). There are also ongoing studies evaluating the use of BVZ in children with newly diagnosed HGG as well, including the currently open COG ACNS-0822 trial. This trial is a randomized Phase II/III trial whereas patients with newly diagnosed HGG will be assigned to one of three chemo-radiotherapy arms, including vorinostat (a histone deacetylase inhibitor) given concurrently with radiation, temozolomide given with radiation, or BVZ given with radiation. All three arms are followed by the same maintenance chemotherapy combination of BVZ and temozolomide (ClinicalTrials.gov, 2010–2012b). This trial is currently accruing patients.

Several biologically targeted agents are under investigation in combination with radiation in newly diagnosed patients and as salvage therapy in children with recurrent disease, including receptor tyrosine kinase inhibitors, histone deacetylase inhibitors, and integrins. One specific targeted therapy that has shown promising pre-clinical data is poly ADP ribose polymerase (PARP) inhibitors. PARP1 is a protein involved in single-strand DNA break repair. Increased PARP1 expression has been observed in HGG as compared to non-neoplastic brain tissue. PARP inhibition potentially enhances sensitivity of tumor cells to DNA damaging agents, including radiotherapy (van Vuurden et al., 2011). Currently, there are ongoing trials in pediatric HGG exploring the use of PARP inhibitors in children with both newly diagnosed and recurrent HGG and DIPG (ClinicalTrials.gov, 2009–2012b, 2012). Many of the data on specific agents is forthcoming, and some experts suggest their role may be best suited as maintenance therapy in the setting of minimal residual disease (Herrington and Kieran, 2009). Another novel approaches to HGG treatment is convection-enhanced delivery (CED). CED utilizes a surgical technique to place a catheter locally and directly into the tumor or tumor resection cavity and directly infuses agents such as chemotherapy, cytotoxic proteins and other biologically targeted agents under a positive pressure gradient (White et al., 2012a,b). Trials utilizing this technique in children with HGG are ongoing (ClinicalTrials.gov, 2006–2012, 2009–2012a).

Immunotherapy has also become an attractive area of research among adult and pediatric HGG. The CNS has long been considered an immunologically privileged site, but it is unclear what limits immunoreactivity within the brain. There has been increasing evidence that during times of CNS insult, there is an increase in the number of lymphocytes within the CNS (Horwitz et al., 1999). In a study evaluating adult HGG in humans, tumor infiltrating lymphocytes (TIL) and regulatory T cells (Treg) were present at a statistically higher frequency as compared to control samples. This increase in lymphocytes was also noted in the peripheral blood of glioma patients as compared to control patients. It is hypothesized that an increase in the Treg cells in the CNS of brain tumor patients may induce a blockade of the natural immune-mediated anti-tumor response. It has been proposed that by countering or depleting these cells, a more vigorous immune-mediated anti-tumor response may be achievable (El Andaloussi and Lesniak, 2006). This hypothesis was evaluated in a murine model and showed prolonged survival in mice by depleting CD4+CD25+ Treg cells (El Andaloussi et al., 2006). Also, by utilizing antibodies that counter this blockade of the immune system, mice with established malignant gliomas achieved 80% long-term survival and evidence of enhanced immunologic response as compared to controls (Fecci et al., 2007). Immune therapies, including vaccine therapies are being increasingly utilized in both adult and children with newly diagnosed and recurrent HGG (Okada et al., 2003, 2007). Many of these trials in pediatrics are still ongoing with results forthcoming (ClinicalTrials.gov, 2005–2010, 2010–2012a).

## BIOLOGY, GENETICS, AND MOLECULAR CHARACTERISTICS

Over the last decade, there is an increasing understanding of the molecular, biologic, and genetic make-up of pediatric HGG. These data have not only helped us to better delineate differing groups of tumors among HGG, but they have allowed development of specific targeted therapies that manipulate our understanding of tumor-related aberrations and oncologic pathways. One of the most common genetic abnormalities in adult HGG is the amplification of epidermal growth factor receptor (EGFR; Libermann et al., 1985; Barker et al., 2001). Although overexpression of the EGFR protein is sometimes seen in pediatric supratentorial HGG, the genetic amplification is quite rare (Libermann et al., 1985; Bredel et al., 1999). There are some data indicating that its expression may be a prognostic marker in specific cohorts of patients treated with EGFR-targeting agents (Geoerger et al., 2011). Mutations in the p53 pathway are a much more common finding in pediatric HGG. Both overexpression of p53 and mutations in the *TP53 *suppressor gene can lead to defects in this pathway and tumorigenesis. Alterations in this pathway have been shown to be prognostically relevant in numerous studies (Pollack et al., 2002; Rood and MacDonald, 2005). Many of the alteration/abnormalities identified in adult HGG such as retinoblastoma gene mutation, amplifications of MYC, MYCN, CDK6, CCND2, deletion of CDKN2C and PTEN mutations are less well understood and overall seemingly less prevalent among children (Broniscer and Gajjar, 2004; Rood and MacDonald, 2005; Jones et al., 2012).

Interestingly, as compared to adult HGG, pediatric HGG have much fewer DNA copy number alterations (Jones et al., 2012). There have been a few consistent chromosomal abnormalities identified in pediatric HGG, including gains at 1p, 2q, and 21q as well as losses noted at 6q, 4q, 11q, and 16q (Rickert et al., 2001; Wong et al., 2006; Bax et al., 2010; Paugh et al., 2010). In particular, as compared to the adult HGG, pediatric HGG seem to possess a statistically higher incidence of gains at 1q and losses at 16q and 4q (Wong et al., 2006; Bax et al., 2010; Paugh et al., 2010; Qu et al., 2010; Schiffman et al., 2010; Barrow et al., 2011; Schwartzentruber et al., 2012). Among the numerous focal genetic alterations elucidated in pediatric HGG, platelet-derived growth factor receptor A (PDGFRA) amplification is by far the most common genomic event identified. This amplification seems to occur most often in older children and may have some prognostic significance (Bax et al., 2010; Paugh et al., 2010; Qu et al., 2010). Another mutation observed in about 10% of pediatric HGG is the V600E point mutation in BRAF (Nicolaides et al., 2011). Interestingly, this seems to be associated with tumors that also possess PDGFRA amplification (Jones et al., 2012). Distinct from the BRAFV600E mutation in many LGG, however, CDKN2A/CDKN2B mutations are more common in these HGG which may help explain why these lesions behave more malignant compared to their LGG counterparts (Jones et al., 2012).

A recent large study evaluated 78 pediatric HGG and DIPG utilizing high-resolution analysis of genomic imbalances using single nucleotide polymorphism microarray analysis. The findings were then compared to data currently understood regarding adult HGG. There were significant differences in copy number alterations that distinguished pediatric from adult HGG (Paugh et al., 2010). PDGFRA was the predominant target of focal amplification in childhood HGG as mentioned above. Specific gene expression analyses identified a possible role for disrupted PDGFRalpha signaling in pediatric HGG. These data again highlighted the growing wealth of information supporting the distinctness between adult and pediatric HGG (Paugh et al., 2010). This group also did not identify a significant number of isocitrate dehydrogenase I (IDHI) mutations in pediatric HGG which have been shown to be quite prevalent and prognostic among adult HGG patients (Horbinski et al., 2009; Labussiere et al., 2010; Paugh et al., 2010). Interestingly, there were some pediatric cases that did cluster with and have signatures more consistent with adult cases suggesting that HGG is a spectrum of diseases that can cross age groups with some subgroups more prevalent in pediatrics and some more common in adults (Paugh et al., 2010). The study also found that there were three distinct subgroups of pediatric HGG identified utilizing unsupervised hierarchical clustering, described as HC1, HC2, and HC3. Analyses of the abnormalities most common in each subgroup revealed that HC1 overexpressed cell cycle regulation genes. HC2 overexpressed neuronal differentiation genes and HC3 overexpressed cellular matrix–receptor interactions and cell adhesion genes (Paugh et al., 2010). Similar to other tumors, like medulloblastoma, for example, identifying specific subgroups within pediatric HGG is becoming increasingly important as an attempt is made to prognosticate and find targeted therapies that are relevant (Northcott et al., 2012; Taylor et al., 2012). Clinically, there already is a distinct difference noted between younger children and older children within pediatric HGG as described previously.

In another large study, the exomes of 48 pediatric HGG were sequenced. Somatic mutations in the H3.3-ATRX-DAXX chromatin remodeling pathway were found in 44% of the samples. Mutations in H3F3A were observed in 31%, which was identified to effect key regulatory post-translational modifications (Schwartzentruber et al., 2012). Mutations in ATRX (alpha-thalassemia/mental retardation syndrome X-linked) and DAXX (death-domain associated protein), were identified in 31% of tumor samples and *TP53* mutations were found in 54% of all cases. *TP53* mutations were found at a higher percent in samples that also had H3F3A and/or ATRX mutations. When the group screened a large separate cohort of gliomas of various grades, they found that the H3F3A mutation appeared to be specific for GBM (Schwartzentruber et al., 2012). They concluded that defects within the chromatin architecture may be critical to pediatric GBM development (Schwartzentruber et al., 2012).

## CONCLUSION: CHALLENGES AND FUTURE DIRECTIONS

Despite the wealth of data regarding the biologic and genetic make-up of pediatric HGG, there remain numerous barriers to understanding the best treatments strategies in children. First, as compared to adults, the number of children with a newly diagnosed HGG is much smaller (CBTRUS, 2012). This makes conducting statistically relevant Phase I and Phase II trials of new agents more difficult (Kauffman, 2000). Also, oral medications are often a challenge with younger children and sometimes specific pediatric formulations are necessary (liquid, for example) which may not be readily available (Abdel-Rahman et al., 2007). The time to develop a trial and obtain appropriate approval in addition to the time necessary to enroll patients and complete a trial often lags behind emerging biologic data. Questions and hypotheses that were novel at the time a trial was conceived may no longer be relevant once a trial is completed. Also, there are limited numbers of available agents and delivery of these agents into the CNS is sometimes fraught with toxicity not typically seen outside of the CNS. Historically, agents are first tested in adults prior to developing Phase I clinical trials in children (Abdel-Rahman et al., 2007). Since it is clear that HGG is distinct in these two populations, simply adopting the adult paradigm may not be the most effect strategy to make advances as witnessed by numerous previous pediatric trials that have utilized this approach (Abdel-Rahman et al., 2007; Gururangan et al., 2010; Cohen et al., 2011).

Separate from the clinical challenges, there are also difficulties within the basic and translational science for pediatric HGG. Unfortunately, some pre-clinical models have been fraught with pitfalls such as tumors that do not mimic human tumor biology, lack of a human microenvironment and poor translation of promising pre-clinical results (Huszthy et al., 2012). It has become increasingly important to begin developing animal models that more closely resemble the distinct variants of pediatric HGG in order to better mimic the human experience. No current animal model is completely identical to the human *in vivo* experience; however, current models are far superior to historic models. For example, historic xenografts derived from chemically induced models or derived from normal glial cells manipulated by media do not reflect the genetic make-up and variability of human glioma. Current techniques of developing xenografts from neurosphere cultures derived from human tumor biopsies more closely recapitulate what is seen in the human, both genetically, phenotypically and clinically (Fomchenko and Holland, 2006; Huszthy et al., 2012). Many of the genetically engineered mouse models also better reflect the invasiveness and genetic make-up of human tumors. These newer models are allowing researchers to begin more carefully assessing what leads to malignant transformation and what characteristics allow cells to become more infiltrative and metastatic (Huszthy et al., 2012). Also, modern labeling techniques have given researchers the ability to more clearly separate the human-derived tumor from the animal host’s cellular compartments and supportive microenvironment allowing for a better understanding of the relationship and interaction between the two (Niclou et al., 2008; Huszthy et al., 2012). Another method utilized to circumvent some of the limitations of animal models is by direct injection of human tumor into the mouse brain. Then, by utilizing advanced imaging techniques, one can assess tumor growth, biologic characteristics, and responses to particular therapies (Jones et al., 2012). Although these models are still fraught with some limitations, the hope is that they will help decrease unnecessary human trials in agents that are not promising or tolerated in the pre-clinical setting.

Advances in technology and basic science methodology has allowed for high-throughput drug screening utilizing both neurospheres and murine models (Houghton et al., 2007; Morton et al., 2007; Whiteford et al., 2007). The Pediatric Pre-clinical Testing Panel (PPTP) is one such endeavor utilizing these techniques to better understand which specific therapeutics may be most suited to further test and develop in pediatric HGG. Currently, the PPTP has numerous primary pediatric HGG samples grown in mice. These models have been utilized in an effort to screen and assess specific agents alone and in combination (Houghton et al., 2007; Kolb et al., 2012; Morton et al., 2012). In order to advance our knowledge of pediatric HGG, we must understand the strengths and weaknesses of each model utilized. It is essential that we understand what specific information we can learn from each model. Only then will we derive a better understanding of pediatric HGG as a whole (Huszthy et al., 2012).

Pediatric HGG is a heterogeneous group of tumors that represent a fraction of pediatric CNS tumors, but unfortunately, they account for a significant amount of morbidity and mortality among pediatric brain tumor patients. These tumors are clearly distinct from their adult counterparts and yet still possess the same very aggressive clinical behavior. Current therapies, including focal radiotherapy utilized in combinations with chemotherapy and biologically targeted agents are not yet sufficient with the far majority of patients succumbing to their disease. The hope is that by obtaining a better understanding of the tumor’s biology and utilizing a variety of pre-clinical models to recapitulate the human tumor biology and microenvironment, we will come one step closer to understanding these tumors more completely. In doing so, there is the potential to target the “right” pathways in a specific tumor and improve patient quality of life and prolong survival.

## Conflict of Interest Statement

The author declares that the research was conducted in the absence of any commercial or financial relationships that could be construed as a potential conflict of interest.
